# The Oral and Skin Microbiomes of Captive Komodo Dragons Are Significantly Shared with Their Habitat

**DOI:** 10.1128/mSystems.00046-16

**Published:** 2016-08-02

**Authors:** Embriette R. Hyde, Jose A. Navas-Molina, Se Jin Song, Jordan G. Kueneman, Gail Ackermann, Cesar Cardona, Gregory Humphrey, Don Boyer, Tom Weaver, Joseph R. Mendelson, Valerie J. McKenzie, Jack A. Gilbert, Rob Knight

**Affiliations:** a BioFrontiers Institute, University of Colorado Boulder, Boulder, Colorado, USA; b Department of Computer Science, University of Colorado Boulder, Boulder, Colorado, USA; c Department of Ecology and Evolutionary Biology, University of Colorado Boulder, Boulder, Colorado, USA; d Graduate Program in Biophysical Sciences, University of Chicago, Chicago, Illinois, USA; e Department of Herpetology, Bronx Zoo/WCS, Bronx, New York, USA; f Denver Zoological Foundation, Denver, Colorado, USA; g Zoo Atlanta, Atlanta, Georgia, USA; h School of Biology, Georgia Institute of Technology, Atlanta, Georgia, USA; i Biosciences Department, Institute for Genomics and Systems Biology, Argonne National Laboratory, Argonne, Illinois, USA; j Departments of Ecology and Evolution and Surgery, University of Chicago, Chicago, Illinois, USA; k Departments of Chemistry and Biochemistry and Computer Science, University of Colorado Boulder, Boulder, Colorado, USA; Michigan State University

**Keywords:** Komodo dragon, SourceTracker, built environment, human microbiome, microbiome

## Abstract

Examining the way in which animals, including those in captivity, interact with their environment is extremely important for studying ecological processes and developing sophisticated animal husbandry. Here we use the Komodo dragon (*Varanus komodoensis*) to quantify the degree of sharing of salivary, skin, and fecal microbiota with their environment in captivity. Both species richness and microbial community composition of most surfaces in the Komodo dragon’s environment are similar to the Komodo dragon’s salivary and skin microbiota but less similar to the stool-associated microbiota. We additionally compared host-environment microbiome sharing between captive Komodo dragons and their enclosures, humans and pets and their homes, and wild amphibians and their environments. We observed similar host-environment microbiome sharing patterns among humans and their pets and Komodo dragons, with high levels of human/pet- and Komodo dragon-associated microbes on home and enclosure surfaces. In contrast, only small amounts of amphibian-associated microbes were detected in the animals’ environments. We suggest that the degree of sharing between the Komodo dragon microbiota and its enclosure surfaces has important implications for animal health. These animals evolved in the context of constant exposure to a complex environmental microbiota, which likely shaped their physiological development; in captivity, these animals will not receive significant exposure to microbes not already in their enclosure, with unknown consequences for their health.

**IMPORTANCE** Animals, including humans, have evolved in the context of exposure to a variety of microbial organisms present in the environment. Only recently have humans, and some animals, begun to spend a significant amount of time in enclosed artificial environments, rather than in the more natural spaces in which most of evolution took place. The consequences of this radical change in lifestyle likely extend to the microbes residing in and on our bodies and may have important implications for health and disease. A full characterization of host-microbe sharing in both closed and open environments will provide crucial information that may enable the improvement of health in humans and in captive animals, both of which experience a greater incidence of disease (including chronic illness) than counterparts living under more ecologically natural conditions.

## Abstract (precis)

Animals, including humans, have evolved in the context of exposure to a variety of microbial organisms present in the environment. Only recently have humans, and some animals, begun to spend a significant amount of time in enclosed artificial environments, rather than in the more natural spaces in which most of evolution took place. The consequences of this radical change in lifestyle likely extend to the microbes residing in and on our bodies and may have important implications for health and disease. A full characterization of host-microbe sharing in both closed and open environments will provide crucial information that may enable the improvement of health in humans and in captive animals, both of which experience a greater incidence of disease (including chronic illness) than counterparts living under more ecologically natural conditions.

## INTRODUCTION

We have long recognized the importance of interactions between animals and their microbial symbionts. Bacteria have facilitated animal evolution, play an important role in animal development and physiology, and perform metabolic processes, such as amino acid synthesis, that their animal hosts cannot perform on their own (1). Fully understanding the role of the host-associated microbiome, however, requires information on how the microbiome is acquired, maintained, and altered through interactions with the environment (both biotic and abiotic) (Tung et al. [2]). On the scale of evolutionary time, vertebrates have only relatively recently begun to regularly interact with artificially built environments, which can have significant differences in temperature, humidity, light, air supply, and microbial exposure compared to the natural environments in which they evolved. It is likely that these differences have notable consequences for vertebrate microbial ecology. Thus, understanding how the degree of microbial sharing between the host and the built environment influences animal health is a priority research area.

Some studies suggest that captivity significantly influences the structure and composition of the host microbiome. Many studies of a wide range of species, including chimpanzees, turkeys, lemurs, and birds, have demonstrated drastic differences in the gut microbiomes of wild animals compared to their captive counterparts (3–7). Similarly, wild-caught salamanders brought into captivity showed a dramatic reduction in skin-associated microbial diversity that was rescued only upon the addition of soil collected from their natural environment into the captive environment (8). Additionally, the diversity of the gut microbiome of wild-caught Atlantic cod decreased when the captive fish were fed an artificial diet, rather than their natural diet (9). Desert woodrats brought into captivity lost 24% of their natural microbes after 6 months in captivity, which the authors suggest may have been due in part to the elimination of their natural diet and in part to changes in host physiology upon introduction into captivity (10). Similarly, the average number of bacterial species cultivated from the mouths of wild Komodo dragons was reported to be 46% higher than the number isolated from captive dragons, although this study leveraged culture-dependent methods, rather than culture-independent, sequencing-based protocols (11).

Characteristic host-environment microbial ecology has also been observed in homes and among the humans and pets living in those homes (12). Human microbial sharing is so specific to the home in which the human lives, that families can be assigned with high accuracy to the home in which they live based on the microbial communities found in the home. Human-associated microbes are also quite pervasive; one family’s microbes overtook their hotel room within 24 h of the family moving into the room (12). Pets also appear to be a conduit for microbe sharing in a house, as couples with dogs share more microbes with each other than couples without dogs (13). The specific effects of closed living on the human microbiome, whether detrimental to health or not, are not entirely clear; however, a recent study of South American Amerindians with no prior documented contact with Western people revealed the highest microbiome diversity ever reported in humans (14). This suggests that lifestyle changes associated with industrialization in human populations—including increasing interactions with closed environments—could have significant effects on microbiome diversity as have been reported in captive-born animals and in wild vertebrates brought into captivity.

The evidence for both vertebrate animals and humans indicates that closed environments not only limit exposure to complex microbial diversity but also promote microbial transfer from the host to the environment, rather than from the environment to the host. Fully characterizing the effects of captivity on host-environment microbial sharing will be key for future studies of vertebrate microbial ecology and may prove instrumental in improving animal husbandry practices. To more thoroughly describe the effects of captivity on host-environment microbiome sharing and how this may affect vertebrate ecology studies, there is a need to examine the microbial ecology of the host-environment interaction in a number of vertebrate species, both in the wild and in captivity. Here we use as a model the captive Komodo dragon (*Varanus komodoensis*), applying 16S rRNA amplicon sequencing to characterize the oral, fecal, skin, and environment-associated microbiomes to answer two main questions: first, is the extent of host-environment microbiome sharing observed for captive Komodo dragons typical of that observed among other vertebrates living in closed environments, and second, is the host-environment microbiome sharing observed among captive Komodo dragons characteristically different from that observed among wild vertebrates? To answer these questions, we explored whether host-environment microbiome sharing in captive Komodo dragons was similar to the pattern observed for humans and pets living in homes and dissimilar to the pattern observed among wild amphibians living in open ecosystems. Together with existing studies, the data suggest that living in closed environments is associated with extensive host-environment microbial sharing. This sharing is likely to be circular in nature—the host contributes microbes to its environment and then, in the absence of significant exposure to microbes from external sources, reacquires those microbes from its environment, only to share them with the environment once again (or vice versa). This may be a radical departure from the microbial communities and exposures which vertebrates cohabitate with and have evolved alongside in the wild, and could have significant effects on health and disease (15).

## RESULTS

In the current study, we obtained skin, saliva, and fecal samples from 37 Komodo dragons in 12 zoos across the United States, in addition to 49 environmental samples from two of these zoos (176 samples total). Deep sequencing of the V4 region of the 16S rRNA gene yielded 5,739,406 high-quality sequences binned into 1,637 operational taxonomic units (OTUs).

### Diversity and composition of the captive Komodo dragon salivary, skin, and fecal microbiota.

**FIG 1:**
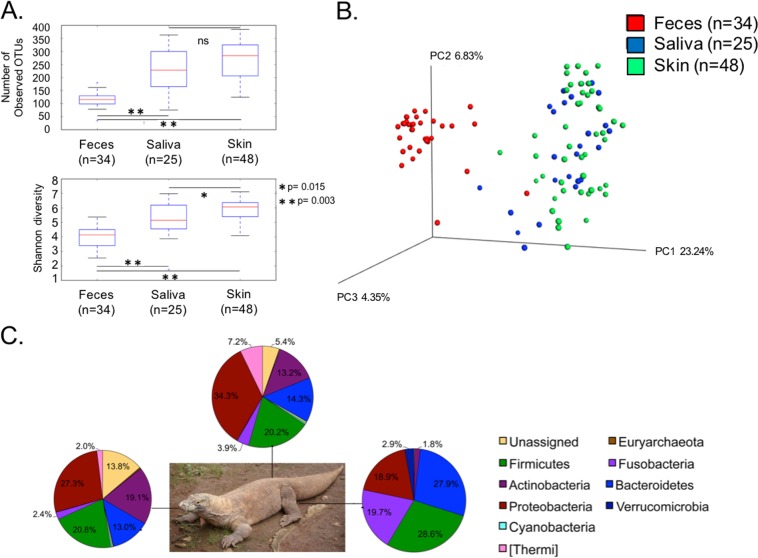
(A) Box-and-whisker plots illustrate the median, maximum, minimum, and first and third quartiles of the distribution of the number of observed OTUs and the Shannon diversity index for Komodo dragon fecal (*n* = 34), saliva (*n* = 25), and skin (*n* = 48) microbial communities. A nonparametric *t* test with Monte Carlo permutations was used to calculate significant differences in diversity between groups. ns, not significant. (B) An unweighted UniFrac-based PCoA plot reveals that fecal microbial communities cluster separately from skin and saliva microbial communities, which are more similar to each other. Principal components 1 to 3 (PC1 to PC3) are shown. (C) Pie charts overlaid onto a Komodo dragon picture illustrate the mean relative abundances of phyla present in Komodo dragon saliva (*n* = 25), skin (*n* = 34), and feces (*n* = 48).

The number of OTUs (*P* = 0.003 [Fig. 1A]) and the Shannon diversity index (*P* = 0.003 [Fig. 1A]) of the Komodo dragon fecal microbial community was significantly lower than that of skin or saliva microbial community. Additionally, although the number of OTUs detected in Komodo dragon skin and saliva did not differ significantly, the Shannon diversity index of the skin microbial community was significantly higher than that of saliva (*P* = 0.015 [Fig. 1A]). A comparison of the unweighted UniFrac distance matrices for each microbial community revealed that Komodo dragon fecal communities clustered separately from skin and saliva communities, which clustered together (Fig. 1B). An adonis significance test revealed that both body site and zoo are significant drivers of sample clustering, though the F statistic associated with body sites was much larger than that associated with zoos (107.305 versus 4.886; *P* = 0.0001; see Table S1 in the supplemental material). Using a single model combining these two variables also produced a significant result (2.093; *P* = 0.0025); however, the F statistic was lower than that associated with either body site or zoo alone (Table S1). There was a nearly 1:1 ratio of *Bacteroidetes* and *Firmicutes* (27.9% and 28.6%, respectively) and of *Fusobacteria* and *Proteobacteria* (19.7% and 18.9%, respectively) in Komodo dragon feces (Fig. 1C), with a lower abundance of *Verrucomicrobia* (2.85%) and *Actinobacteria* (1.79%). Komodo dragon skin and saliva communities were more similar to each other than either was to feces, with both containing at least 13% *Actinobacteria* as well as the phylum Thermi (7.2% and 2.0%, respectively), which was not detected in feces (Fig. 1C). Additionally, core fecal, skin-, and saliva-associated microbiota were identified (Table S2). Eight OTUs were found in both the skin and salivary core microbiomes, four OTUs were found in both salivary and fecal core microbiomes, and five OTUs were found in both the skin and fecal core microbiomes.

10.1128/mSystems.00046-16.5 Table S1  Performance of the leave-one-out random forest classifier when classifying samples from individual Komodo dragon enclosures. The number of environmental samples belonging to individual Komodo dragon enclosures (identified by animal name) that were correctly assigned to their enclosure of origin by the classifier are listed, with the corresponding class errors. Download Table S1, DOCX file, 0.1 MB. Copyright © 2016 Hyde et al. 2016 Hyde et al. This content is distributed under the terms of the Creative Commons Attribution 4.0 International license.

10.1128/mSystems.00046-16.6 Table S2  Numbers of core OTUs found in subsets of the entire Komodo dragon cohort, from 50 to 100%, for saliva, skin, and feces. Download Table S2, DOCX file, 0.1 MB. Copyright © 2016 Hyde et al. 2016 Hyde et al. This content is distributed under the terms of the Creative Commons Attribution 4.0 International license.

### Comparing the diversity and composition of the Komodo dragon salivary, skin, and fecal microbiomes to their captive environmental surfaces.

**FIG 2:**
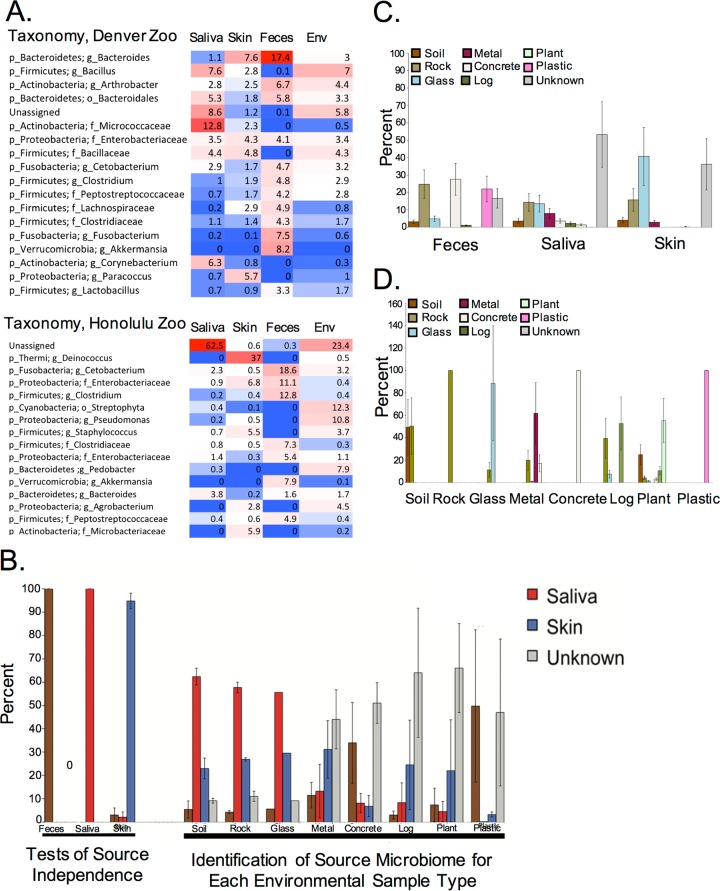
(A) Heat maps illustrate the percent abundances of the most abundant genera (all OTUs taxonomically classified to the same genus were collapsed into a single genus summary) present in saliva, skin, feces, and environmental (Env) samples collected from the Denver and Honolulu zoos. The deepest taxonomic classification achieved is listed for each genus. The heat map colors indicate percent abundance (red [high abundance] to blue [low abundance]). (B) Komodo dragon SourceTracker analysis reveals that the microbial communities of many environmental sample types are sourced from skin, saliva, and feces rather than unknown sources (i.e., not from Komodo dragon skin, saliva, or feces). Data are plotted as the means ± standard errors of the means (error bars) of samples from Denver and Honolulu zoo Komodo dragons. (C) SourceTracker analyses with Komodo dragon fecal, salivary, and skin samples designated as sinks and environmental samples designated as sources reveals that a variety of environmental sources, rather than a single environmental source, contribute to the microbial communities of Komodo dragon feces, saliva, and skin. Unknown sources (i.e., not the environments sampled from the Komodo dragon enclosures) also contribute about 40% or more of the microbial community of saliva and skin samples (only 20% of fecal samples). (D) Independence tests reveal that about half of the environmental samples are not independent from other environmental samples. Data are the means ± standard errors of the means of Denver and Honolulu Komodo dragon and environmental samples.

To determine how much of the Komodo dragon’s microbiome is shared with its environment (or vice versa) and whether and how specific the environment is to the dragon, we obtained matched dragon-environment samples from a subset of zoos (Denver and Honolulu). Overall, the diversity of environmental samples was significantly higher than that of skin, saliva, and feces (*P* = 0.006 [see Fig. S1A in the supplemental material]), and the diversity of all environmental materials was relatively similar (with the exception of water [Table S3]). Consistent with these results, environmental samples clustered closer to skin and saliva samples than to fecal samples by principal-component analysis (PCoA) (Fig. S1B). In terms of taxonomic composition and abundance, environmental microbiomes appeared most similar to salivary and skin microbiomes from the phylum down to the genus level in both the Denver and Honolulu zoo cohorts (Fig. 2A and Fig. S2 Although the number of environmental samples from Honolulu Zoo dragons was limited both in number (three) and type (soil or plant material) compared to the number and range of environmental samples collected from the Denver Zoo, similar trends were observed in both zoos. Most notably, the phylum *Actinobacteria* (mainly *Corynebacteriaceae* and *Micrococcaceae*) was detected in skin, saliva, and environmental samples but at a much lower relative abundance in feces. The phylum *Fusobacteria* (*Fusobacteriaceae*) was also present at similar abundances in skin and in the environment but at a much higher relative abundance in feces.

10.1128/mSystems.00046-16.1 Figure S1  (A) Box-and-whisker plots illustrate the median, maximum, minimum, and first and third quartiles of the distribution of the number of observed OTUs and the Shannon diversity index for Komodo dragon fecal, saliva, skin, and environmental microbial communities. A nonparametric *t* test with Monte Carlo permutations was used to calculate significant differences in diversity between groups. (B) Unweighted UniFrac-based PCoA reveals that environmental samples cluster closer to Komodo skin and saliva samples than to Komodo fecal samples. Download Figure S1, PDF file, 0.2 MB. Copyright © 2016 Hyde et al. 2016 Hyde et al. This content is distributed under the terms of the Creative Commons Attribution 4.0 International license.

10.1128/mSystems.00046-16.7 Table S3  The number of OTUs and Shannon diversity index of each environmental material sampled from captive Komodo dragon enclosures at a rarefaction depth of 3,210 sequences per sample. Data are presented as the means (± standard deviations) of each alpha diversity metric calculated on 10 rarefaction iterations. Download Table S3, DOCX file, 0.1 MB. Copyright © 2016 Hyde et al. 2016 Hyde et al. This content is distributed under the terms of the Creative Commons Attribution 4.0 International license.

10.1128/mSystems.00046-16.2 Figure S2  Stacked bar charts illustrate the mean relative abundances of bacterial taxa detected in Komodo dragon saliva, skin, feces, and environmental samples collected from the Denver and Honolulu zoos at the phylum (A and B), class (C and D), order (E and F), and family (G and H) levels. Download Figure S2, PDF file, 0.1 MB. Copyright © 2016 Hyde et al. 2016 Hyde et al. This content is distributed under the terms of the Creative Commons Attribution 4.0 International license.

### Specificity and extent of Komodo dragon-environment microbiome sharing.

We applied SourceTracker to samples from the Denver Zoo Komodo dragons to determine which dragon microbiome sources (saliva, feces, and skin) contributed to the dragon environment. The microbiomes of items in the Denver Komodo dragons’ enclosures were largely sourced from Komodo dragon salivary, skin, and fecal samples (Fig. 2B), with unknown sources comprising less than 50% of the microbial communities of most environmental sample types. Additionally, skin, saliva, and fecal communities were distinct from one another in a SourceTracker independence test (Fig. 2B), suggesting that any skin, saliva, or fecal communities detected on environmental materials actually came from the dragon’s skin, mouth, or feces. Further supporting this point—at least in the context of saliva—several bacterial taxa found in the mouths of the Komodo dragons studied here, including *Staphylococcus*, *Corynebacterium*, *Pseudomonas*, and *Bacteroides*, have previously been reported in the mouths of captive Komodo dragons (11, 16). This suggests that environmental microbes designated as sourced from the Komodo dragon’s oral cavity likely actually do come from the mouth and not any other source. Repeating this analysis on the entire Komodo dragon cohort yielded comparable results (see Fig. S3 in the supplemental material). The nature and extent of host-microbiome transfer to environmental objects varied with sample type; for example, Komodo dragon saliva was the main source of the microbial communities detected in soil and on rock and glass, while Komodo dragon skin was the main source of the microbial communities detected on metal (Fig. 2B. Performing SourceTracker analyses with Komodo dragon samples designated as sinks and environmental samples designated as sources revealed that the microbial communities of Komodo dragon fecal, saliva, and skin samples are sourced from a variety of environmental materials, each contributing 30% or less of the microbial community (Fig. 2C and D). There is no one environmental material that contributes more than any other material to Komodo dragon feces or saliva; however, Komodo skin microbial communities are sourced majorly from glass and unknown sources (each ~40%).

10.1128/mSystems.00046-16.3 Figure S3  SourceTracker analysis revealed that the microbial communities of the majority of environmental sample types are sourced from Komodo dragon skin, saliva, and feces rather than unknown sources (i.e., not Komodo dragon skin, saliva, or feces), as seen in panel A, and that each Komodo source was independent from each other source, as seen in panel B. Data are the averages ± standard errors of the means of the entire Komodo dragon cohort (12 zoos). Download Figure S3, PDF file, 0.1 MB. Copyright © 2016 Hyde et al. 2016 Hyde et al. This content is distributed under the terms of the Creative Commons Attribution 4.0 International license.

Using leave-one-out Random Forest classification on samples collected at the Denver Zoo, we see that the microbial communities populating individual Komodo dragon enclosures are significantly different. The classifier achieved a baseline error to observed error ratio of 4, indicating that it was able to correctly assign an environmental sample to the enclosure from which that sample was obtained. The classifier was very successful on environmental samples obtained from the enclosures of two Denver Komodo dragons that had been at the zoo for an extended period of time, with only one sample being incorrectly classified, while the class error was higher when classifying environmental samples collected from the enclosures of two dragons that had recently arrived from Europe (Table 1).

**TABLE 1:** Performance of the leave-one-out random forest classifier when classifying samples from individual Komodo dragon enclosures^a^

Komodo dragon	No. of environmental samples from individual enclosures correctly assigned to:				Class error (%)
	Anika	Kristika	Raja	Tujah	
Anika	4	2	0	0	33.30
Kristika	3	3	0	0	50
Raja	0	0	11	1	8.30
Tujah	0	0	0	15	0

a The number of environmental samples belonging to individual Komodo dragon enclosures (identified by animal name) that were correctly assigned to their enclosure of origin by the classifier are listed, with the corresponding class errors. The estimated error was 0.15385, the baseline error was 0.61538, and the baseline error/estimated error ratio was 4.00.

To further assess host-environment microbiome sharing, in both closed/captive and open/wild environments, we additionally performed SourceTracker analyses on two previously published data sets—a wild amphibian skin-environment microbiome data set (17) and a human-pet-house microbiome data set (12)—and compared them to the Komodo dragon data set. As previously shown, humans and their pets contribute a large amount of their microbiomes to their living environments (12), similarly to the patterns we observed with captive Komodo dragons. However, while Komodo dragon microbiome sources (skin, saliva, and feces) were found to be distinct sources, we did not observe this level of source independence when applying the SourceTracker independence test to the human/pet data set (see Fig. S4A in the supplemental material). Designating human and pet samples as sinks and house surfaces as sources revealed that the microbial communities of human and pet samples are sourced from a variety of environmental materials (Fig. S4B), which was also observed for captive Komodo dragons (Fig. 2).

10.1128/mSystems.00046-16.4 Figure S4  (A) SourceTracker independence tests reveal that dogs and cats are independent sources, while humans are less independent from their pets. (B) Designating humans and pets as sinks and home surface samples as sources reveals that a variety of home surfaces contribute to the microbial communities of humans and their pets. Unknown sources (i.e., not the home surfaces) also contribute a small percentage (less than 20%) of the human and pet microbial communities. Download Figure S4, PDF file, 0.6 MB. Copyright © 2016 Hyde et al. 2016 Hyde et al. This content is distributed under the terms of the Creative Commons Attribution 4.0 International license.

**FIG 3:**
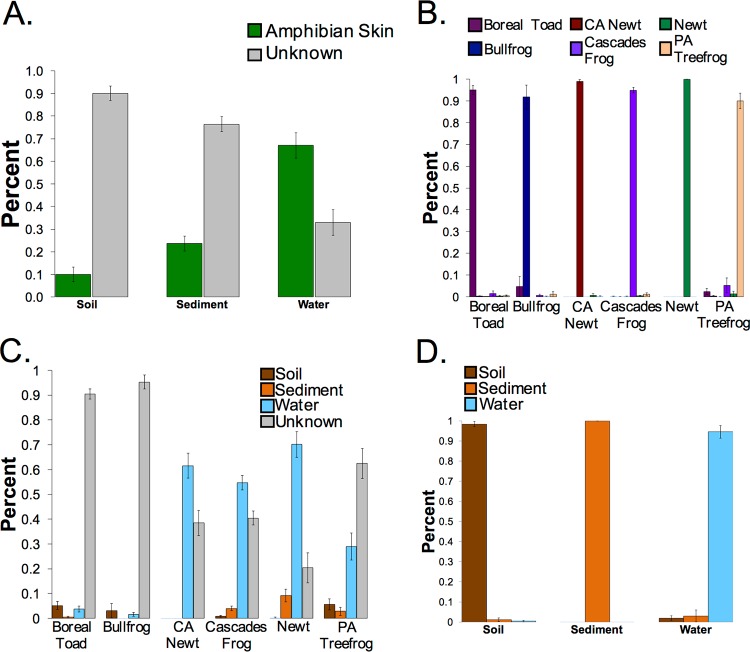
(A) Amphibian SourceTracker analysis reveals that water is the only sample type that obtains a notable amount of its microbial community from amphibian skin; unknown sources (i.e., not amphibian skin) are the main microbiome contributors to soil and sediment. (B) Independence tests reveal that amphibian skin is independently specific to species. (C) Designating environment the source and amphibian skin the sink reveals that water is the only environmental type that contributes largely to the microbial communities on amphibian skin, with unknown sources also largely contributing to the amphibian skin microbiome. (D) Independence tests reveal that each environment type is also independent from each other environment type. Data are the means ± standard errors of the means (error bars).

Host-environment microbiome sharing between amphibians and their living environment was not as extensive as that observed among captive Komodo dragons and their enclosures or humans and pets and their homes. More than 75% of soil and sediment microbial communities were obtained from unknown microbiome sources; however, the identified “source” for 75% of water microbial communities was amphibian skin (Fig. 3A). Each source (here defined as individual amphibian species) was highly independent from each other source (Fig. 3B). Defining amphibian skin as a sink and environmental samples as sources, water was identified as a major source of the microbes on the skin of most species; nevertheless, at least 20% of the microbial community on the skin of all species was contributed by unknown sources (Fig. 3C). Soil, sediment, and water were all confirmed to be independent sources (Fig. 3D).

**FIG 4:**
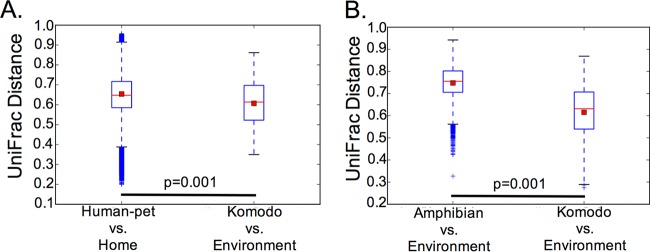
Box-and-whisker plots illustrate the UniFrac distance between Komodo dragon and Komodo dragon environment samples and human/pet and home samples (A) and amphibian and amphibian environment samples (B).

Unweighted UniFrac distances between Komodo dragon and Komodo dragon environment samples and human/pet and home samples were smaller than the distance between amphibian and amphibian environmental samples (*P* = 0.01 and Bonferroni-corrected *P* = 0.06) (Fig. 4), demonstrating that the closed environments analyzed here are associated with more-similar microbial communities between the environment and the organisms present in that environment than are the open environments.

## DISCUSSION

This study represents the first description of the captive Komodo dragon skin and fecal microbial communities, filling an important knowledge gap, as previous studies have focused on the Komodo dragon salivary microbiome (11, 16). Importantly, this study also characterizes the captive environment for these animals, providing information regarding the animal-environment interactions that occur in captivity. These data are foundational for developing translational approaches to exploring Komodo dragon microbial ecology, particularly regarding host-environment microbiome sharing in captivity, and moving this experience toward that which occurs in the wild.

Captive Komodo dragons share their skin, salivary, and fecal microbiomes extensively with their environments. Different sharing patterns were observed with different environmental sample types, and this variation was likely due in part to the nature and extent of the animal’s interaction with each item in its environment. As expected, the microbial communities of environmental materials with which the animal extensively interacts (soil and rock) are mainly sourced from Komodo dragon microbiomes. The sample types in which half of the environmental microbial community came from unknown, non-Komodo dragon sources, were concrete, logs, plant materials, and plastic. The unknown microbial sources in the case of the logs/plants may be native plant microbial communities or chloroplasts, which we have often seen at high abundance in other internal projects characterizing plant microbiomes (18); additionally, it is unknown how much time the Komodo dragons interact with these materials or how often the plastic in the dragon’s enclosure was cleaned by the caretakers, all of which will affect how much Komodo dragon microbial material is detected on these items. Importantly, applying a test of independence on designated microbial sources within SourceTracker revealed that each Komodo dragon microbial community (saliva, skin, and fecal) was distinct from each of the others, with saliva classifying as saliva, skin classifying as skin, and feces classifying as feces. This indicates that the salivary, skin, and fecal microbial communities detected on environmental objects were likely sourced from Komodo dragon saliva, skin, and feces. As described above, the presence of bacterial taxa in the saliva of the captive Komodo dragons studied here that have been previously observed in captive Komodo dragons (11, 16) adds further evidence that Komodo dragon salivary bacterial communities are serving as actual sources rather than sinks. Unfortunately, to date, no studies have characterized Komodo dragon skin or fecal microbial communities, so it is impossible to compare the results obtained here with existing knowledge.

Komodo dragon enclosures were specific to the animals residing in them, as the microbial community of an individual enclosure could discriminate that enclosure from all other enclosures at the same zoo. Whether or not Komodo dragons in the wild are characterized by such individually specific microbiomes is unknown. Wild Komodo dragons are solitary animals most of the time (19). They do, however, come together to reproduce and sometimes congregate at large prey kills to eat (19), activities that can certainly facilitate extensive sharing of microbes. In captivity, social interactions, minimal as they are in the wild, are even less frequent; they typically occur only during breeding (J. R. Mendelson III, personal observation), and although juveniles may be housed together, adult captive Komodo dragons live virtually solitary lives. Therefore, it is difficult to extrapolate the microbial ecology of the captive Komodo dragon to its wild counterpart, particularly in terms of host-host microbiome sharing. Further studies on host-host and host-environment sharing in wild Komodo dragons will prove crucial for determining the significance of host-environment microbiome sharing in Komodo dragon health and whether the effect of the microbiome is as strong as other known environmental factors. For example, nonavian reptiles (including Komodo dragons) in captivity suffer poor health due to lack of environmental exposure to UV-B radiation, and these health issues can be prevented by maintaining them in outdoor enclosures or installing lighting that includes UV-B spectra and supplementing the diet of the animals with vitamins. On the other hand, microbes do play a role in the health of some reptiles—juvenile herbivorous turtles and lizards suffer from poor health if they are not exposed to adult fecal matter (20–22); in these animals, engaging in coprophagy to “seed” the microbiome appears to be a key aspect of normal health. While coprophagy has been observed among varanid lizards (*Varanus scalaris*) under extremely stressful conditions, for example, ground cover loss and prey depletion after a fire (23), it appears to be a rare behavior among monitor lizards. Whether captivity could encourage monitor lizards, such as Komodo dragons, to participate in coprophagy and whether this behavior would prove beneficial to the animal in the captive situation remain unknown.

The extensive and specific Komodo dragon-environment microbiome sharing we report here mirrors that observed among humans and their homes, as reported by Lax et al. (12), who were able to use the microbial communities present in a home to predict which family lived in that home. This is in contrast to host-environment microbial associations that have been observed in wild populations. For example, using a field-collected data set that compares the skin microbiome of wild amphibians and their environment (17), we demonstrated that the amphibian skin microbiome represents a subset of the environmental microbes that occur in the water. Similar results were also observed by Walke et al. (24), who studied two different amphibian species living in a single pond and concluded that amphibian skin may select for certain environmental microbes. Furthermore, Loudon et al. (8) demonstrated that when wild amphibians (salamanders, in this case) are removed from their soil-based hibernacula and taken into captivity in sterile plastic containers, the diversity of bacteria on their skin diminishes notably through time, unless they are housed in captivity with a soil substrate taken from their natural habitat. Similarly, Becker et al. (25) demonstrated that when Panamanian golden frogs are maintained in captivity long term with natural soil substrate, they maintain 70% of the bacterial OTUs found on wild counterparts. Thus, for amphibians, environmental source bacteria appear to play a critical role in the assembly and maintenance of skin microbiome diversity. More studies characterizing the microbiome dynamics associated with the movement of an animal from the wild into captivity will likely prove instrumental in informing captive animal husbandry.

While the precise implications of the nature of host-environment microbiome sharing in captivity compared to that in the wild are unknown, an increased incidence of disease in urbanized humans as well as in captive animals compared to their wild counterparts suggests that the consequences may be significant (15). As humans have become increasingly urban, we have seen an increase in many disease conditions, including allergies, asthma, and several chronic conditions (26). The decreased exposure to the outdoors and other microbe-rich locations together with the increased indoor lifestyles (working and living) and obsessive cleaning habits that characterize Western culture today have led to the formulation of the hygiene hypothesis (27), which posits that a lack of exposure to beneficial microbes present in the environment leads to the increase in disease that we have seen in the past century. Captive animals, which live in enclosed environments not equivalent to their normal environments in the wild, experience diseases that are associated with or worsened by the captive environment, including gastric ulcers (vervet monkeys [28]), intestinal stricture (green sea turtles [29]), and end-stage renal disease (polar bears [30]). As we and others have shown, human houses and Komodo dragon zoo enclosures represent closed locations characterized by possibly circular microbial sharing with less microbial input from outside environments than that received by animals and humans living and working predominantly outdoors. Therefore, indoor-dwelling, captive animals such as the Komodo dragons studied here are likely not exposed to significant microbial diversity other than that already inside their enclosures.

All of these studies together suggest that a lack of environmental microbial exposure could be associated with several health issues observed among not only industrialized human populations but also in captive animals. Introducing commensal microbial species obtained from wild animals or environmental material to captive animals and their environments, adopting a cohousing strategy where appropriate (or rotation of housing where not), or introducing animals housed primarily indoors to outdoor enclosures for part of the day could alleviate or lessen the severity of some captivity-associated health complications. Cohousing would certainly introduce the animal to microbes other than its own. In this study, two dragons that had recently arrived at the Denver Zoo (Anika and Kristika) were housed in wired enclosures (unlike the two long-time Denver Zoo residents), facilitating contact through the partition and microbiome sharing, and it was the enclosures of these two dragons that were less easily discriminated from one another (Table 1). Additionally, although Komodo dragon microbiome sources (skin, saliva, and feces) proved to be independent from one another, human microbiome sources were not characterized by this same individuality, proving to be a mix of human and pet microbiomes (see Fig. S4 in the supplemental material). Animals housed together will similarly potentially share individual-animal-specific microbial communities with each other, exposing those animals to more varied microbial diversity compared to solitary animals. Additionally, both humans and their pets do go outside and are therefore exposed to more microbial diversity than captive animals housed individually in indoor enclosures. We saw evidence of this in that the distance distributions between human/pet and home samples indicated higher UniFrac distances than between captive Komodo dragons and their environments, suggesting that individual housing with minimal to no outdoor exposure may be associated with more extensive and/or specific host microbiome sharing with the built environment.

More studies need to be done to further define the effect of captivity on the microbiome and implications for disease, and how to utilize the microbiome to improve health in captive animals. For example, humans may represent an important source of beneficial or harmful bacteria for captive animals, and studying aquarium and zoo animals that interact more with humans than do Komodo dragons (i.e., educational outreach animals) will be important to fully understand the breadth of host-environment microbiome sharing in captive animals. It is only by characterizing the microbiomes of both wild and captive animals and by identifying important changes in environmental and even social interactions that have detrimental effects on the microbiome that we will be able to understand the precise connection between captivity, the microbiome, and health and disease.

## MATERIALS AND METHODS

### Sample collection.

The skin, saliva, and feces of captive Komodo dragons (housed individually) were sampled at twelve U.S. zoos (Zoo Atlanta [two dragons], Bronx Zoo [three dragons], Denver Zoo [five dragons], Fort Worth Zoo [four dragons], Gladys Porter Zoo [one dragon], Honolulu Zoo [six dragons], Houston Zoo [four dragons] Jacksonville Zoo and Gardens [two dragons], Los Angeles Zoo [five dragons], ABQ BioPark Zoo [two dragons], Virginia Aquarium [two dragons], and Woodland Park Zoo [two dragons]). All samples were collected using sterile, double-headed swabs. Skin was swabbed by firmly rubbing the swab across the designated area of skin for at least 10 s. Saliva was collected by either allowing the dragon to tongue flick the swab, catching drool, or by inserting the swab slightly into the mouth of the animal. Fecal material was collected by touching the surface of the swab heads to the surface of the feces just enough to turn the swab head the color of the feces. Environmental materials at two zoos were also swabbed. Environmental objects at the Denver Zoo swabbed included rock, metal, plastic, glass, soil, wood, and plant material, while only soil and plant matter were sampled at the Honolulu Zoo. All animals were sampled under IACUC protocol 1203.04 at the University of Colorado Boulder and a Blood and Tissue Use protocol that permitted the receipt of fecal, salivary, skin, and environmental samples. Protocols for collection were additionally approved at the Fort Worth Zoo, Bronx Zoo, Denver Zoo, Los Angeles Zoo, Woodland Park Zoo, and Zoo Atlanta.

Komodo dragons were sampled during the summer or early fall months (June to October) of 2012 by their zoo caretakers. Each zoo received the same set of sampling instructions to ensure consistency of sample collection across all zoos. Immediately after collecting the sample, the swabs were frozen at −20°C before shipment (on dry ice) to the University of Colorado Boulder for DNA extraction and sequencing.

### Bacterial genomic DNA extraction and 16S rRNA gene amplification and sequencing.

DNA extraction, amplicon generation, and amplicon preparation for sequencing were performed by the protocols recorded in reference 31 and can be found on the Earth Microbiome Project (EMP) web page (http://www.earthmicrobiome.org/emp-standard-protocols/).

Bacterial genomic DNA was extracted from fecal, saliva, skin, and environmental samples using the PowerSoil DNA isolation kit (MoBio Laboratories, Carlsbad, CA). PCR amplification of the V4 region of the 16S rRNA gene was performed similarly to the method of Caporaso et al. (31). Briefly, each sample was amplified in triplicate and combined. PCR mixtures contained 13 µl of MoBio PCR water, 10 µl of 5 Prime hot master mix, 0.5 µl each of the forward and barcoded reverse primers (515f [f stands for forward] and 806r [r stands for reverse]; 10 µM final concentration), and 1.0 µl of genomic DNA. The reaction mixtures were held at 94°C for 3 min (denaturation), with amplification proceeding for 35 cycles, with 1 cycle consisting of 45 s at 94°C, 60 s at 50°C, and 90 s at 72°C, with a final extension of 10 min at 72°C to ensure complete amplification.

After amplification, DNA concentration was quantified using the Picogreen double-stranded DNA (dsDNA) reagent in 10 mM Tris buffer (pH 8.0). A composite sample for sequencing was created by combining equimolar ratios of amplicons from the individual samples, followed by ethanol precipitation to remove any remaining contaminants and PCR artifacts. The composite sample was sequenced using the Illumina HiSeq platform at the BioFrontiers Institute Next-Generation Genomics Facility at the University of Colorado Boulder. The data are publically available at the European Bioinformatics Institute (EBI) (study ERP016252) as well as at https://qiita.ucsd.edu (study identification [ID] number 1747).

### Data analysis.

The data were prepared and analyzed using the QIIME pipeline (32) version 1.8, and the analysis is publicly available as an IPython notebook (http://nbviewer.ipython.org/gist/josenavas/c8ec4bce222636fe79a5). The forward read was quality filtered and demultiplexed according to the following parameters: no ambiguous bases allowed, only one mismatch in the barcode sequence allowed, and a minimum Phred quality score of 20. Quality filtering resulted in 5,739,406 high-quality sequences total. The quality-filtered sequences were then clustered using either the closed-reference OTU picking workflow against the August 2013 release of the Greengenes database (33) with a sequence identity of 97% and uclust (34) as the underlying clustering algorithm, or the open-reference workflow (35), also using the August 2013 release of the Greengenes database, a sequence identity of 97%, and uclust as the clustering algorithm. Using the closed-reference workflow, only 72.7% of sequences clustered against the Greengenes database; therefore, all further analyses were performed on the open-reference OTU-picked data set, which recovered 95.2% of sequences. The open-reference OTU table was further quality controlled by filtering out OTUs not represented by at least 0.005% of all reads in the data set. Additionally, all samples with less than 3,323 reads per sample were removed from the OTU table, resulting in a total of 151 samples used in downstream analyses.

### (i) Alpha diversity and associated statistical analyses.

The number of observed species and the Shannon diversity index were calculated for each sample in the data set with at least 3,000 reads. The OTU table corresponding to these samples was randomly subsampled 10 times at a sequencing depth from 10 sequences per sample to 3,210 sequences per sample in steps of 100 sequences. The alpha diversity metrics were then calculated on the resulting rarefied OTU tables. Statistical analyses were performed to determine significant differences in alpha diversity between body site and body site plus environment within the Komodo dragon cohort. Statistical analyses were performed using a nonparametric *t* test with Monte Carlo permutations (999) to calculate the *P* value.

### (ii) Beta diversity and associated statistical analyses.

The UniFrac distance (36) was calculated on a rarefied OTU table of 3,323 sequences per sample. Principal-component analysis (PCoA) was applied to the resulting distance matrix, and plots were generated using Emperor software (37). Beta diversity analyses performed on combined Komodo dragon-amphibian and Komodo dragon-human data sets followed the same protocol, with the Komodo dragon-amphibian data set rarefied to 5,870 sequences per sample and the Komodo dragon-human data set rarefied to 5,357 sequences per sample. To analyze the significance of sample groupings, anosim (nonparametric, 999 permutations) and permanova (nonparametric, 999 permutations) tests were performed on the UniFrac distance matrices. We further compared distances by performing two sample *t* tests (999 permutations) to determine whether the distance distributions between Komodo dragons and their environment differed from those between humans and pets and their environment or amphibians and their environment. We also examined the individuality of four Komodo dragon enclosures at the Denver Zoo using the randomForest R package implemented with the QIIME workflow. The rarefied OTU table containing only environmental samples was the input for a leave-one-out random forest analysis. Group classifications were performed on the enclosures as indicated by the individual dragon residing in each enclosure.

### (iii) SourceTracker analyses.

SourceTracker (38) is a tool that uses a Bayesian model jointly with Gibbs sampling to quantify the number of taxa that a set of source environments contributes to a sink environment. We have applied SourceTracker to define which Komodo dragon microbiomes (skin, saliva, and feces) are shared with the environment, specifically by defining Komodo dragon saliva, skin, and feces as microbial “sources” and Komodo dragon environmental materials as microbial “sinks.” We also applied SourceTracker to amphibian samples from a previously published study in which skin and environmental (water, soil, and sediment) samples were collected from five amphibian species (17) and additionally human, pet, and house samples (12). The reverse SourceTracker analyses (defining host samples as sinks and environmental samples as sources) were also performed on all three data sets. Any OTUs that were not present in at least 1% of samples were removed from the OTU table before implementing SourceTracker to ensure that useful OTUs were provided to the algorithm (39). Additionally, leave-one-out source sample predictions were run in parallel for each SourceTracker analyses to test the independence of each source. While taxonomic composition analyses compared Denver and Honolulu zoo samples, only Denver zoo samples were utilized for SourceTracker analyses, as both the variety and number of environmental samples collected from the Denver Zoo were more extensive than those collected from the Honolulu Zoo.

### (iv) Comparison of captive Komodo dragon soil samples with North and South American soil samples.

To compare the microbial communities of captive Komodo dragon soil to “wild” environmental soil, we combined quality-filtered sequences belonging to captive Komodo dragon soil samples with those belonging to soil collected from various ecosystems in North and South America (40, 41). We performed the open-reference OTU-picking workflow, as described above, and created interactive PCoA plots using Emperor software. We assessed sample clustering using the anosim and permanova statistical tests.
