# Supplementary material for: The Oral and Skin Microbiomes of Captive Komodo Dragons Are Significantly Shared with Their Habitat
Source: mSystems. 2016 Aug 2;1(4):e00046-16. doi: 10.1128/mSystems.00046-16 (PMC5069958; doi:10.1128/mSystems.00046-16)
Supplement: Table S1 [file sys004162043st5.docx]

| **Comparison: Body Site** | | | **Comparison: Zoo** | | | **Comparison: Body Site + Zoo** | | |
| --- | --- | --- | --- | --- | --- | --- | --- | --- |
| **F statistic** | **R^2^** | **p-value** | **F statistic** | **R^2^** | **p-value** | **F statistic** | **R^2^** | **p-value** |
| 107.305 | 0.58596 | 0.0001 | 4.886 | 0.12006 | 0.0001 | 2.093 | 0.10286 | 0.0025 |

**Table S1.** The statistical test adonis was applied to the unweighted UniFrac distance matrix to assess whether either body site (fecal n=34, saliva n=25, sebum n=48) or zoo (Zoo Atlanta n=17, Denver Zoo n=15, Honolulu Zoo n=8, Houston Zoo n=10, Los Angeles Zoo n=11, Rio Grande Albuquerque n=6, Virginia Aquarium n=11, Woodland Park Zoo n=5) significantly drive differences between samples. These two variables were additionally combined in a single model. Two zoos that were not represented by at least five samples (Bronx Zoo, n=3 and Jacksonville Zoo and Gardens, n=4) were first removed from the dataset.
