# Supplementary material for: The Oral and Skin Microbiomes of Captive Komodo Dragons Are Significantly Shared with Their Habitat
Source: mSystems. 2016 Aug 2;1(4):e00046-16. doi: 10.1128/mSystems.00046-16 (PMC5069958; doi:10.1128/mSystems.00046-16)
Supplement: Table S2 [file sys004162043st6.docx]

| Saliva | | Skin | | Stool | |
| --- | --- | --- | --- | --- | --- |
| % dragons | # OTUs | % dragons | # OTUs | % dragons | #OTUs |
| 50 | 68 | 50 | 100 | 50 | 74 |
| 55 | 59 | 55 | 81 | 55 | 58 |
| 60 | 51 | 60 | 65 | 60 | 43 |
| 65 | 35 | 65 | 47 | 65 | 36 |
| 70 | 26 | 70 | 34 | 70 | 33 |
| 75 | 19 | 75 | 26 | 75 | 25 |
| 80 | 13 | 80 | 19 | 80 | 17 |
| 85 | 6 | 85 | 14 | 85 | 13 |
| 90 | 4 | 90 | 8 | 90 | 8 |
| 95 | 3 | 95 | 4 | 95 | 2 |
| 100 | 1 | 100 | 0 | 100 | 0 |

**Table S2.** The number of core OTUs found in subsets of the entire Komodo dragon cohort, from 50-100%, for saliva, skin, and feces.
