# Supplementary figures and images for: The Oral and Skin Microbiomes of Captive Komodo Dragons Are Significantly Shared with Their Habitat
Source: mSystems. 2016 Aug 2;1(4):e00046-16. doi: 10.1128/mSystems.00046-16 (PMC5069958; doi:10.1128/mSystems.00046-16)

A.

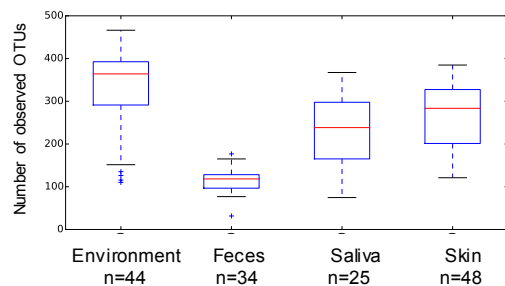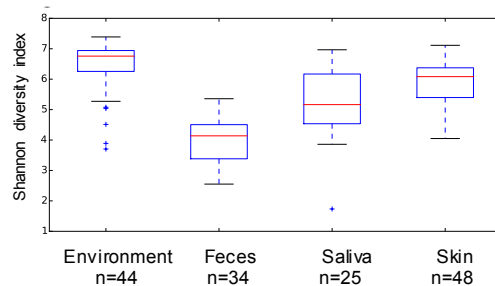

B.

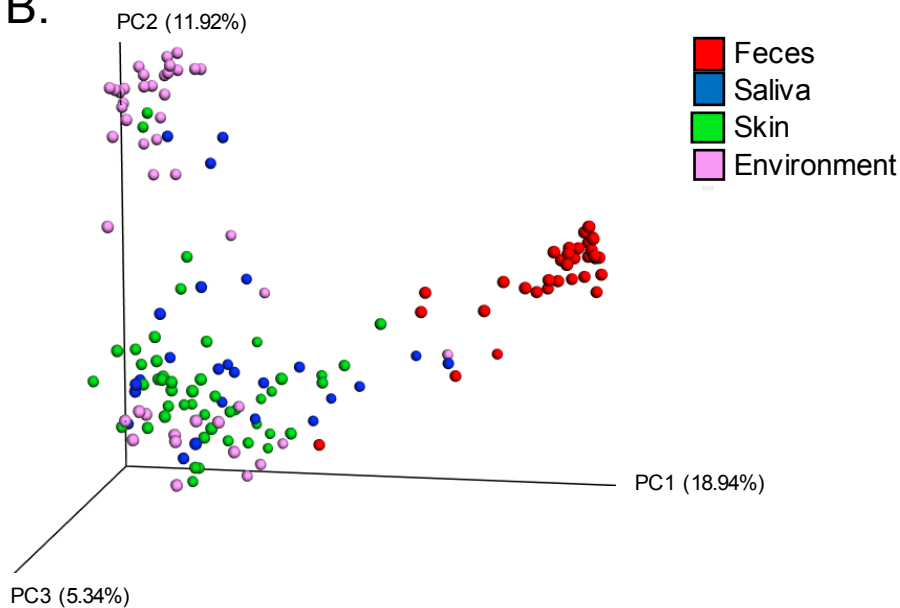

Supplement: Figure S1 [file sys004162043sf1.pdf]

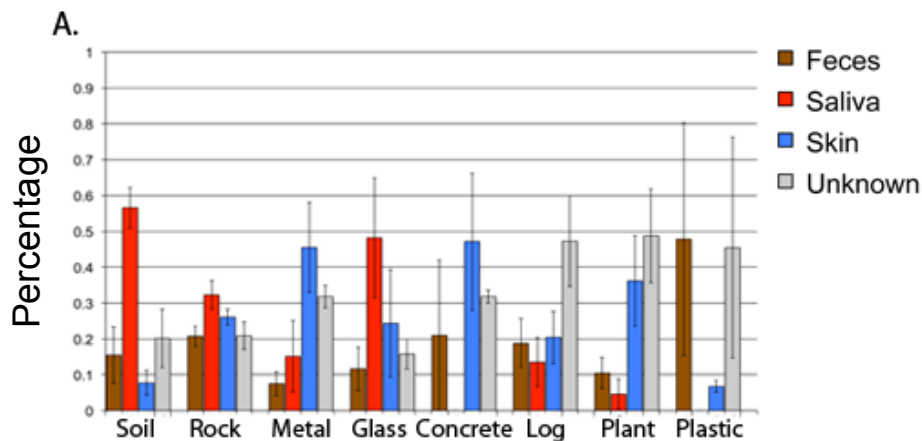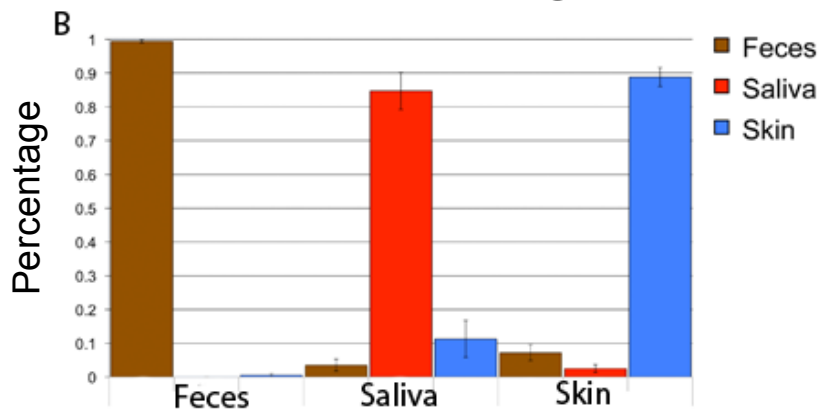

Supplement: Figure S3 [file sys004162043sf3.pdf]

A.

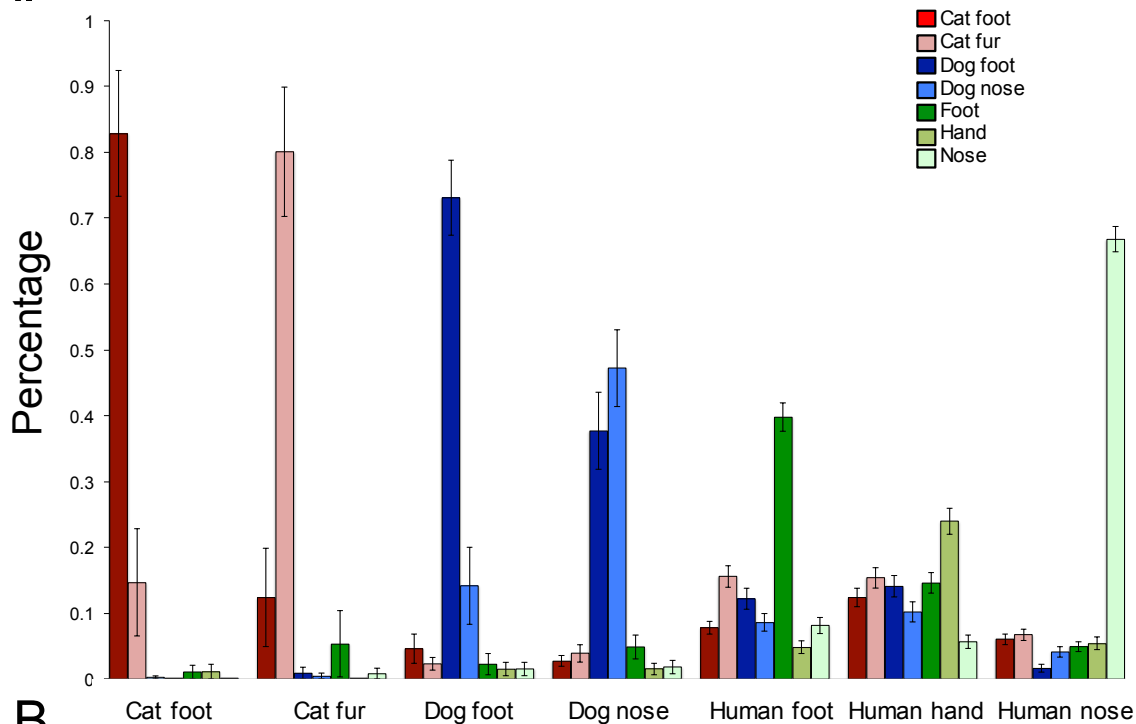

B.

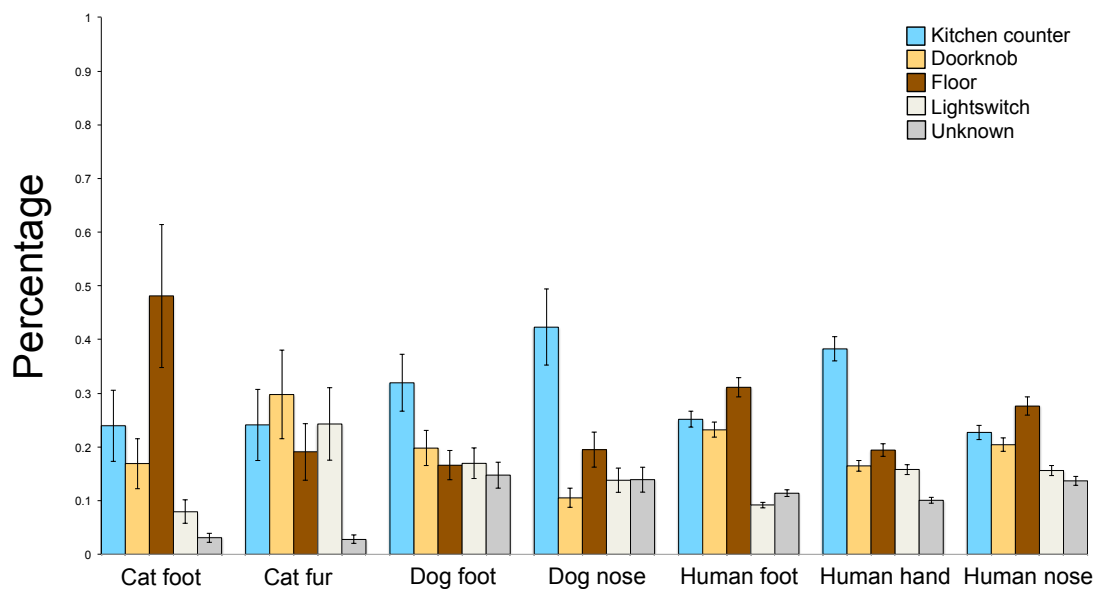

Supplement: Figure S4 [file sys004162043sf4.pdf]
