# Supplementary material for: The Oral and Skin Microbiomes of Captive Komodo Dragons Are Significantly Shared with Their Habitat
Source: mSystems. 2016 Aug 2;1(4):e00046-16. doi: 10.1128/mSystems.00046-16 (PMC5069958; doi:10.1128/mSystems.00046-16)
Supplement: Table S3 [file sys004162043st7.docx]

|  | Number of OTUs | Shannon diversity index |
| --- | --- | --- |
| Metal | 385.3 (+/- 2.12) | 7.13 (+/- 0.01) |
| Rock | 365.7 (+/- 3.22) | 6.75 (+/- 0.01) |
| Concrete | 358.3 (+/- 4.93) | 7.01 (+/- 0.03) |
| Soil | 351.0 (+/- 6.62) | 6.63 (+/- 0.02) |
| Plastic | 341.0 (+/- 6.92) | 6.03 (+/- 0.04) |
| Plant material | 331.5 (+/- 1.83) | 6.26 (+/- 0.01) |
| Glass | 322.5 (+/- 3.47) | 6.65 (+/- 0.02) |
| Water | 131.8 (+/- 3.28) | 4.94 (+/- 0.02) |

**Table S3.** The number of OTUs and Shannon diversity index of each environmental material sampled from captive Komodo dragon enclosures at a rarefaction depth of 3210 sequences per sample. Data are presented as the mean (+/- standard deviation) of each alpha diversity metric calculated on ten rarefaction iterations.
