# Supplementary material for: The Oral and Skin Microbiomes of Captive Komodo Dragons Are Significantly Shared with Their Habitat
Source: mSystems. 2016 Aug 2;1(4):e00046-16. doi: 10.1128/mSystems.00046-16 (PMC5069958; doi:10.1128/mSystems.00046-16)
Supplement: Figure S2 [file sys004162043sf2.pdf]

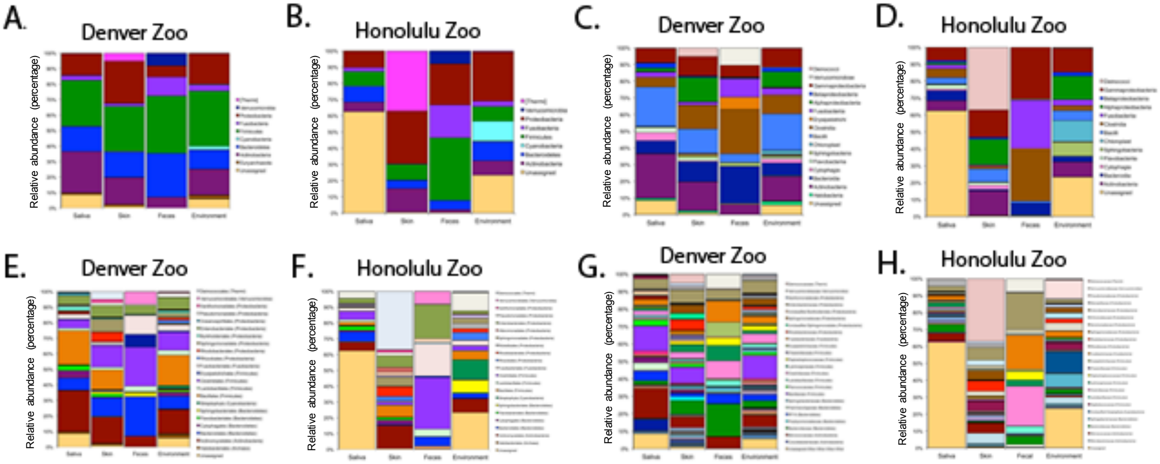
A.
Denver Zoo
B.
Honolulu Zoo
C.
Denver Zoo
D.
Honolulu Zoo
E.
Denver Zoo
F.
Honolulu Zoo
G.
Denver Zoo
H.
Honolulu Zoo
Relative abundance (percentage)
